# No time for pending confirmation of invasive fungal disease in critically ill COVID-19 patients—think empirical treatment

**DOI:** 10.1186/s13054-020-03307-5

**Published:** 2020-09-29

**Authors:** Anne-Lise Bienvenu, Nathalie Bleyzac, Jean-Christophe Richard, Gilles Leboucher

**Affiliations:** 1 Service Pharmacie, Groupement Hospitalier Nord, Hospices Civils de Lyon, 103 grande rue de la Croix-Rousse, 69004 Lyon, France; 2 ICBMS CNRS 5246, Campus Lyon-Tech La Doua, Université de Lyon, Lyon, France; 3 Service de réanimation médicale, Groupement Hospitalier Nord, Hospices Civils de Lyon, Lyon, France

To the Editor,

In a recent study, Wang et al. [1] reported invasive pulmonary aspergillosis in patients with coronavirus disease 2019 (COVID-19), thus claiming for an early intervention with bronchoscopy and the importance of obtaining evidence of fungal microbiology in patients with severe/critical COVID-19. We fully agree on this point considering that invasive fungal infections (IFI), mostly invasive pulmonary aspergillosis, are increasingly reported in COVID-19 patients admitted in intensive care units (ICU); coronavirus-associated pulmonary aspergillosis (CAPA) was demonstrated to affect up to 30% of ventilated patients with COVID-19 [2]. Nevertheless, few issues should be addressed.

First, CAPA diagnosis is challenging. Questions remain if COVID-19 patients have a true invasive aspergillosis or are just colonized with *Aspergillus* in regards with serum galactomannan negativity [3]. Nevertheless, an independent association between CAPA and 30-day mortality was recently demonstrated using a multivariate logistic regression model among intubated patients [2], although a causal link remains to date unproven.

Second, the use of corticosteroids or anti-IL6 antibody in addition to standard of care is proposed for critically ill patients with COVID-19. But corticosteroids are well-known risk factors for IFI and identified as a negative outcome predictor of invasive aspergillosis [4], whereas IFI were reported in patients treated with anti-IL6 antibody [5]. Thus, it is expected that incidence of IFI may increase with the more extensive use of corticosteroids or other immunomodulating therapies, and this, mostly in patients with risk factors for CAPA such as older age, initial antibiotic usage of beta-lactamase inhibitor combination, and chronic obstructive pulmonary disease (COPD) [1].

In this context, it is urgent to consider the opportunity of empirical use of antifungals without waiting for the final evidence of fungal microbiology, in case of a clinical suspicion of IFI. This is especially true for critically ill COVID-19 patients receiving immunomodulating therapies. Certainly, it will be most of the time an off-label use of antifungals in ICU patients, but what a prize to win under the emergency conditions of COVID-19 pandemic. Antifungal stewardship programs should support the implementation of such strategies, including the discontinuation of antifungals with negative mycological results and the impact assessment of antifungal use in terms of efficacy, safety, drug interactions, and resistance emergence.

## Data Availability

Not applicable.

## Abbreviations

CAPA: Coronavirus-associated pulmonary aspergillosis
COVID-19: Coronavirus disease 2019
ICU: Intensive care units
IFI: Invasive fungal infections
COPD: Chronic obstructive pulmonary disease
